# MIF: functions in brain and glioblastoma

**DOI:** 10.18632/oncotarget.18489

**Published:** 2017-06-12

**Authors:** Shigeki Ohta, Yutaka Kawakami, Hideyuki Okano

**Affiliations:** Division of Cellular Signaling, Institute for Advanced Medical Research, Keio University School of Medicine, Tokyo, Japan

**Keywords:** MIF, CHD7, neural stem cells, brain tumor-initiating cells

Neural stem/progenitor cells (NSPCs) are multipotent precursors present in the mammalian embryonic and adult brain. These cells are capable of undergoing proliferation and self-renewal, as well as of generating neurons, astrocytes, and oligodendrocytes, and are thus potentially useful in drug screening and cell based therapeutic approaches targeting neurodegenerative diseases. Induced pluripotent stem cell derived-NSPCs (iPSC-NSPCs) represent an attractive alternative to human fetal brain derived-NSPCs for use in such research and applications [1]. A number of soluble factors and signaling molecules have been shown to participate in the regulation of NSPCs; however, it is still important to deepen the understanding of NSPCs in terms of their developmental origins, which have direct implications for their therapeutic potential. In a previous study, we identified MIF (Macrophage migration Inhibitory Factor) as a novel factor contributing to NSPC proliferation and maintenance of stemness [2]. MIF is a pro-inflammatory factor involved in many diseases including atherosclerosis and rheumatoid arthritis, as well as a pro-tumor factor. MIF has been reported to induce Arginase 1 production by pro-tumor myeloid-derived suppressor cells (MDSCs) in the tumor immune microenvironment (TME) [3]. MIF also plays a contrastingly beneficial role through its activation of AMP-activated protein kinase (AMPK) in an ischemia model. Thus, MIF appears to be a pleiotropic factor that functions in a pathological context-dependent fashion; however, the function of MIF in the CNS disorders have remained largely unknown, which prompted us to investigate MIF function in NSPCs.

MIF is secreted from mouse and human NSPCs cultured as neurospheres, and serves as a ligand for the CD74/CD44 complex and a non-cognate ligand for CXCR2 and CXCR4. We have observed the expression of MIF receptors in mouse cultured neurospheres, indicating possible autocrine activity. In mouse NSPCs, we have also identified downstream signaling molecules (e.g., Erk, Stat3, AMPK) regulated by MIF. Additionally, *in vitro* studies by our group have shown that MIF contributes to NSPC cell migration. Subsequently, we also determined that Sox6, a SOXD transcription factor, is regulated by MIF in mouse NSPCs, and contributes to their cell proliferation and stemness maintenance, mediated in part by Stat3 signaling [4]. Chromatin Helicase-DNA-binding protein 7 (CHD7), chromatin remodeling factor, is also regulated by MIF in mouse NSPCs and human embryonic stem cell-derived NSPCs (hES-NSPCs), and also contributes to cell proliferation and maintenance of stemness [5]. CHD7 has been reported to contribute to neural crest induction [6] and alternations in the CHD7 gene are causative of CHARGE syndrome (a complex genetic disorder characterized by coloboma, heart defect, atresia choanae, retarded growth and development, genital hypoplasia, and ear anomalies/deafness) and autism spectrum disorders. Interestingly, the Whi (CHD7 point mutation) mouse shows a reduction in proliferative intermediate progenitor cells (IPCs) in the embryonic mouse brain, leading to defects in neurogenesis [5]. Another group has also reported that CHD7 regulates neurogenesis in a study using adult mouse NSPCs [7]. We are currently seeking to identify other signaling molecules downstream of MIF in hES-NSPCs using RNA sequencing and miR arrays, and have recently found that TPT1 (Tumor Protein, Translationally-Controlled 1) is a downstream of MIF in NSPCs (unpublished data).

One principal goal of our group is the elucidation of mechanisms that regulate the activity of brain tumor-initiating cells (BTICs). Glioblastoma, the most common form of primary malignant brain tumor, is a fatal malignancy that is now thought to involve a heterogeneous cellular population initiated and maintained by a subpopulation of BTICs. MIF expression in BTICs is higher than in non-BTICs and human astrocytes [8]. In tumor-derived neurosphere culture *in vitro*, BTICs cultured from glioblastoma patient tumors can be expanded longer than non-BTICs. MIF gene knockdown in BTICs results in both reduced cell proliferation and increased apoptosis *in vitro*. In a human BTIC mouse xenograft models, MIF gene silencing showed therapeutic effects. This study also reported the intracellular localization of MIF in glioma cells, in which MIF directly binds p53, thereby regulating cell proliferation and apoptosis [8]. These important findings notwithstanding, the intracellular function of MIF in glioblastoma remain largely unknown. Whether MIF contributes to astrocyte de-differentiation into BTICs through p53 dysregulation is another question of particular interest. Detailed studies of the intercellular functions of MIF in glioblastoma are thus warranted. A recent study by our group has shown that MIF-regulated CHD7 regulates the proliferation of BTICs [5 and unpublished data]. An *in silico* analysis using the Cancer Genome Atlas (TCGA) data revealed that expression of the CHD1, CHD4, CHD7, and CHD 9 genes is upregulated in glioblastoma, while that of the CHD3 and CHD5 genes is downregulated. More intensive studies of the CHD family, which consists of multi-complexes (e.g., the NuRD-complex) in BTICs, may lead to the identification of epigenetic drugs targets for glioblastoma. In ongoing work, our group is seeking to identify the precise mechanisms underlying MIF signaling, with a focus on BTIC epigenomics. Notably, MIF expressed in human iPSCs regulates cell proliferation (unpublished data), lending additional support to a role for MIF in promoting cell proliferation in many stem cell types, including NSPCs, BTICs, and iPSCs. It remains unknown whether MIF utilizes distinct signaling pathways in different stem cell types. Further mechanistic analyses of MIF signaling will undoubtedly yield new insights and may pave the way for the development of new drug candidates targeting BTICs in glioblastoma.
